# Investigating the Role of Primary Cilia and Bone Morphogenetic Protein Signaling in Periodontal Ligament Response to Orthodontic Strain In Vivo and In Vitro: A Pilot Study

**DOI:** 10.3390/ijms252312648

**Published:** 2024-11-25

**Authors:** Emily R. Moore, Anna Konermann

**Affiliations:** 1 Department of Developmental Biology, Harvard School of Dental Medicine, Boston, MA 02115, USA; 2 Department of Orthodontics, University of Bonn, 53111 Bonn, Germany

**Keywords:** bone morphogenetic proteins, mechanotransduction, orthodontic tooth movement, periodontal ligament cells, primary cilia, strain

## Abstract

Periodontal ligament (PDL) cells are crucial for mechanosensation and mechanotransduction within the PDL, yet the role of primary cilia in orthodontic force transmission has not been examined. While bone morphogenetic protein (BMP) signaling significantly influences ciliary function, its effect on cellular responses to mechanical stress has not been investigated. This study aims to investigate whether primary cilia and BMP signaling are involved in the periodontal ligament’s response to orthodontic tooth movement and the resultant mechanical strain. To visualize primary cilia, human PDL cells were cultured on glass-bottom dishes for five days, with a subset fixed daily, followed by immunostaining with anti-acetylated α-tubulin and Alexa Fluor 568 and imaging using a fluorescence microscope under 405 nm and 561 nm laser excitation. Human PDL cells were grown on Bioflex^®^ culture plates and subsequently exposed to static tensile strains of 2.5%, 5%, 10%, 20%, on a FX-6000T™ Tension System for 24 h. RT-qPCR was performed to evaluate changes in expression of primary cilia via *Ift88* expression, mechanotransduction via *Cox2* expression, and *BMP* signaling-related genes. Histological specimens from orthodontically loaded and control human premolars were investigated for primary cilia and BMP signaling using immunohistochemistry and confocal microscopy. Primary cilia were observed in PDL cells from day one, with their incidence and length increasing over time alongside cell density. BMP signaling components, including upregulated genes such as *Bmp7* (10.99–14.97 fold), *Alk2* (3.19–5.45 fold), and *Bmpr2* (1.64–8.40 fold), consistently responded to strain, while *Cox2* and *Ift88* showed differential regulation depending on strain intensity. In vivo, orthodontic movement activated BMP signaling and increased primary cilium incidence in the PDL. These findings indicate the potential role of primary cilia and BMP signaling in the mechanosensitivity of PDL cells under orthodontic forces. Further studies are required to understand the complex mechanotransduction mechanisms and role of these components in cellular adaptation during orthodontic tooth movement.

## 1. Introduction

The periodontal ligament (PDL) is a specialized fibrous connective tissue located between tooth root cementum and alveolar bone. It plays a critical role in sensing mechanical forces, in addition to providing structural support to the teeth, facilitating nutritional supply, maintaining tissue homeostasis, and supporting repair processes [1]. PDL cellular networks withstand physiological strains induced by mastication, as well as the pronounced compressive and tensile forces associated with orthodontic tooth movement (OTM), leading to displacement of teeth within the alveolar bone [2,3]. The specific orthodontic stresses initiate a cascade of cellular and molecular events, culminating in accelerated remodeling of the PDL and adjacent bone, triggering sterile inflammation [4,5]. Analogous to physiological bone remodeling, both tensile and compressive forces are exerted during this highly coordinated process. Specifically, osteoclasts facilitate bone resorption on the compression side, and bone formation is driven by osteoblasts on the tension side [6]. However, excessive compressive forces during OTM can result in a pathological autoimmune tissue resorption referred to as external root resorption (ERR) [7]. This adverse phenomenon initiates with the degradation of the superficial root cementum and may subsequently progress to an irreversible stage of resorption. Such adverse side effects can also manifest from orthodontic forces conventionally considered clinically safe, as well as idiopathically in cases where no discernible causative factors are identified [8]. PDL cells, constituting the predominant cellular population within the PDL, can directly perceive mechanical stimulation and convert these physical cues into intracellular signaling cascades through the process known as mechanotransduction. These signaling responses result in secretion of signaling molecules that orchestrate the aforementioned remodeling processes [9]. Although mechanically driven repair is critical for maintaining tissue homeostasis and preventing autoimmunological conditions, the intricate mechanisms through which these cells sense and transduce physical signals into transcriptional responses remain unknown.

A promising candidate for mediating mechanotransduction within the PDL is the primary cilium, potentially orchestrating cellular responses to biomechanical forces encountered during OTM. Primary cilia are solitary, nonmotile microtubule-based organelles present on most cells in the body. They project from the cell surface and function as chemo- and mechanosensory entities, facilitating the transduction of extracellular cues into intracellular responses. Primary cilia formation and function are fundamentally dependent on intraflagellar transport (IFT), a specialized process involving the transport of macromolecules essential for maintenance of this dynamic organelle [10]. Deletion of various IFT components, especially Ift88, results in disruption of primary cilia formation and function [11]. The primary cilium is considered a signaling amplifier because it is highly enriched with signaling receptors such that the cilium can sense and respond to external stimuli that other regions of the cell cannot detect. The variety of receptors present also enables the cilium to mediate multiple signaling pathways simultaneously. Thus, primary cilia act as sensory antennae to detect a wide range of extracellular signals, including signaling molecules and physical perturbations. In the context of mechanotransduction, the primary cilium is believed to bend and open stretch-activated ion channels located at its base where tension is maximal [12,13]. It is well established that primary cilia are pivotal in regulating cellular proliferation, differentiation, and signaling, and they exert a profound impact on tissue formation and homeostasis, including craniofacial structures [10,14]. However, the occurrence and functional role of cilia within the PDL remain inadequately explored to date, with the existing literature limited to a small number of studies. Current evidence indicates that primary cilia are present on approximately 70% of PDL cells, exhibit stage- and region-specific morphologies, are implicated in tooth development and may play a role in mechanotransduction [15,16,17,18]. The random orientation of primary cilia within the ligament provides additional evidence for their mechanosensory function, as the axonemes are strategically positioned to detect physical movement from all directions [14].

While existing research has delineated the critical role of BMP signaling in the formation, maintenance, and repair of periodontal tissues, its precise involvement in the processes of tooth movement and mechanical loading of PDL cells remains unexplored [19]. An expanding body of evidence underscores the complex interplay between ciliary mechanotransduction and bone morphogenetic proteins (BMPs) [20,21]. At this, signaling pathways associated with BMPs are anticipated to modulate ciliary function in a force-dependent manner, with distinct effects observed under high versus low mechanical forces [22].

To date, the roles of primary cilia and BMP signaling in the PDL cell response to orthodontic movement remains unexamined, whether conducted in vivo or in vitro. The scope of this study was to fill this gap by exploring the role of primary cilia and BMP signaling in the mechanotransductive response of human PDL cells to OTM. The research hypothesis is that primary cilia act as mechanosensors, modulating BMP signaling to regulate cellular processes during OTM. The investigation examines the presence, structural dynamics, and functional significance of primary cilia in PDL cells exposed to varying mechanical strains, alongside the transcriptional regulation of key BMP signaling molecules. A mechanotransduction pathway is proposed in which primary cilia enhance BMP receptor activation, thereby initiating intracellular signaling cascades that influence PDL cell behavior. This investigation aims to elucidate the mechanistic link between primary cilia and BMP signaling, offering insights into the cellular response to mechanical forces and providing potential therapeutic targets to mitigate complications such as ERR in orthodontics.

## 2. Results

**Figure 1:**
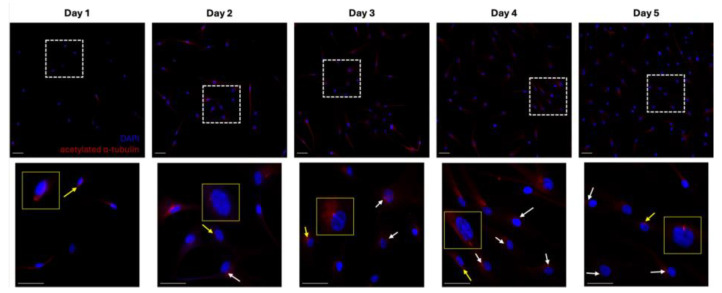
Primary cilia are present in primary human PDL cell cultures. The panels display primary cilia in isolated PDL cells cultured for 1 to 5 days. Primary cilia were detected using an antibody against acetylated α-tubulin (red, indicated by white arrows). Cell nuclei were depicted with DAPI staining (blue). Structures indicated by the yellow arrow are enlarged in the yellow box. Images were collected at 20× magnification. Sample size: *n* = 1 per timepoint.

Previous studies have indicated the presence of PDL cells, aiming this investigation to firstly confirm their existence in primary cultures established from human samples. Analysis revealed that primary cilia were present in PDL cells as early as the first day of cell culture. A temporal increase in the prevalence of primary cilia was noted, and the incidence and length of primary cilia appeared to increase in positive correlation with cell density (Figure 1). This observation aligns with findings in other cell types, where primary cilia exhibit increased prominence as cell density rises. More importantly, this finding substantiated the existence of dynamic primary cilia within the PDL cell preparations, thereby validating these isolations as viable models for investigating mechanotransduction in vitro.

**Figure 2:**
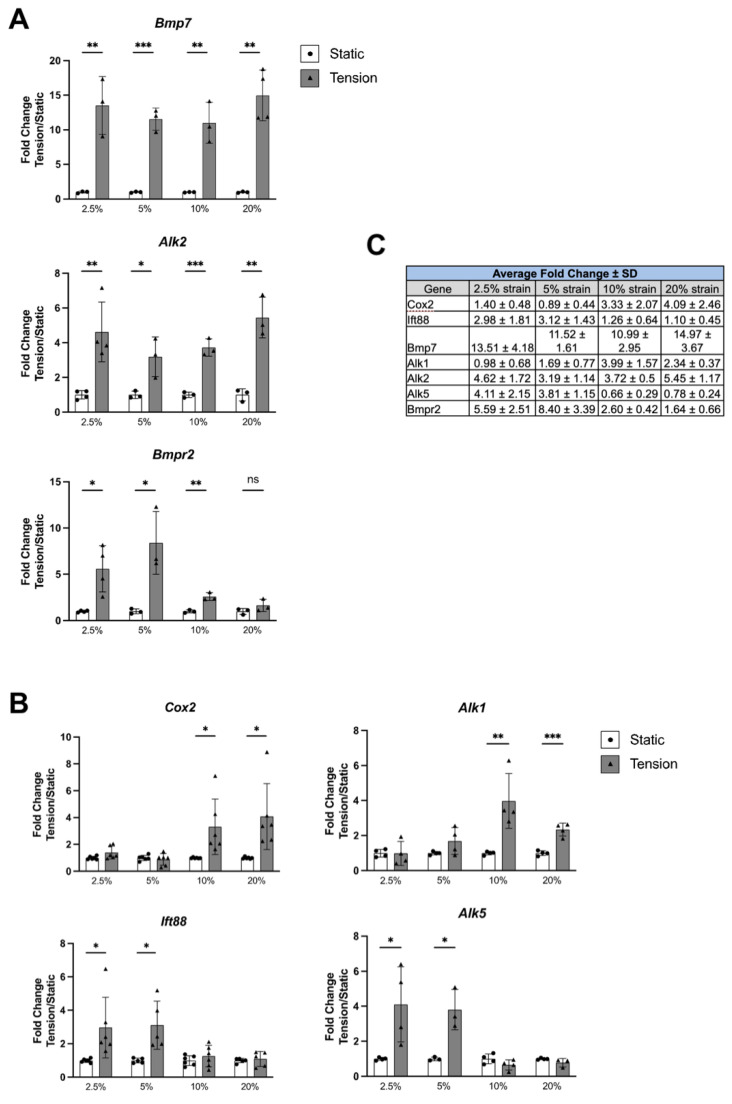
Transcriptional changes in BMP, cilia, and mechanosensation-associated genes in primary human PDL cells exposed to tensile strain. (**A**) The genes Bmp7, Alk2, and Bmpr2 were consistently upregulated across all magnitudes of tensile strain. (**B**) Cox2, a marker for cellular response to physical stimulation, and Alk1, a type I BMP signaling receptor, were exclusively upregulated at high strain levels of 10% and 20%. In contrast, Ift88, a primary cilia marker, and ALK5, another type I BMP signaling receptor, showed increased expression only under low strain intensities of 2.5% and 5%. Fold changes were measured between control cells (static, white columns) and cells exposed to varying tensile strains (tension, gray columns) as indicated on the x-axis in percent (%). Values are represented as fold changes ± SEM compared to static controls and mRNA expression was normalized to the housekeeping gene GAPDH. Statistical significance is denoted as follows: * *p* < 0.05, ** *p* < 0.01, *** *p* < 0.001. (**C**) Average fold changes of the genes presented as mean ± SD. Sample size: *n* = 3–4 per group.

Subsequently, the expression of components of the BMP signaling pathway and the key regulator of primary cilia formation and function, Ift88, was assessed in isolated PDL cells. Investigations confirmed the expression of Ift88 and all core components of the BMP pathway, except for the type I receptor Alk4 (Table 1). To further analyze the cellular response, primary PDL cells were subjected to tensile strain mimicking OTM, and changes in mRNA expression were evaluated after 24 h of stimulation. Multiple strain intensities were tested as previous studies indicate that cellular responses may vary with strain magnitude. Expression of Cox2, a marker for cellular response to physical stimulation, was increased significantly only at 10% and 20% strain magnitudes (Figure 2A,C). Conversely, the primary cilia marker Ift88 showed elevated expression only under low strains of 2.5% and 5%. Overall, changes in BMP signaling components varied greatly with strain magnitude and donor; however, trends could be observed that are summarized in Table 1. Some components, such as Alk1 and Alk5, exhibited statistically significant magnitude-dependent trends similar to those observed with Cox2 and Ift88 (Figure 2B,C). Like Cox2 and Alk1, Bmp9 expression generally increased with low strain magnitudes (Table 1). Expression of Smad5, Bmp4, Alk7, Acvr2a, Acvr2b, and Tgfbr2 exclusively increased when cells were subjected to the highest strain of 20%. Smad1 expression was not altered at low strain but significantly decreased at 10% and 20% strains. Interestingly, expression of the ligand Bmp7, type I receptor Alk2, and type II receptor Bmpr2 was consistently increased across strain magnitudes (Figure 2B), suggesting that Bmp7, Alk2, and Bmpr2 are candidate components in the cellular response to mechanical loading of the PDL.

The table summarizes trends in mRNA expression of BMP signaling superfamily components (including signaling transcription factors, ligands, and receptors), and known markers for the cellular response to mechanical stimulation (Cox2) and primary cilia (Ift88). Components that are not expressed in PDL cells are highlighted in pink. Gray shading indicates no observed change between stretched and static control samples. Three genes that were consistently upregulated at all levels of tensile strain (Bmp7, Alk2, and Bmpr2) are highlighted in yellow. Sample size: *n* = 3–4 per group.

**Figure 3:**
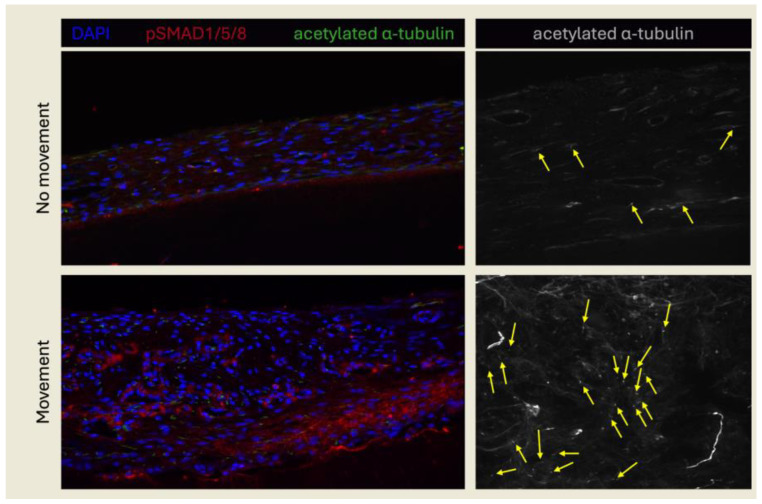
Activation of BMP signaling and primary cilium incidence in the PDL with OTM. Teeth subjected to OTM (movement) and static controls (no movement) were prepared for histological analyses. BMP signaling activation was visualized using an antibody against pSMAD1/5/8 (red) and primary cilia were detected using an antibody against acetylated α-tubulin (green in left panel, white in right panel). Cell nuclei were depicted with DAPI staining (blue). Examples of primary cilia are marked by yellow arrows. Images were acquired at 40× magnification (left panel) and 60× magnification (right panel). Sample size: *n* = 3 per group.

The in vitro results of this study provide support for the involvement of primary cilia and BMP signaling in PDL cells. To determine whether these components are involved in OTM, primary cilia and BMP signaling were visualized in the PDL of human teeth under static or movement conditions in vivo. In control teeth without movement, minimal BMP signaling activity was observed in the PDL (Figure 3). However, BMP signaling was highly activated in response to OTM compared to controls. Analysis of primary cilia presence revealed that not all cells within the PDL exhibited a primary cilium, irrespective of whether the tooth was subjected to movement or remained stationary. Primary cilium incidence drastically increased in the regions where BMP signaling activity was detected compared to the PDL in static teeth and regions of the PDL in moved teeth where BMP signaling was absent. These findings suggest that BMP signaling and primary cilia play a role in OTM and may also participate in PDL mechanotransduction in vivo. Additionally, the co-localization of BMP signaling activity with enhanced ciliogenesis implies a potential interaction between these mechanisms.

## 3. Materials and Methods

This study was conducted in adherence to the ethical principles outlined in the World Medical Association Declaration of Helsinki. Informed consent was secured from all individual human donors contributing experimental material. Additionally, the study underwent independent review and received approval from the Ethical Committee of the University of Bonn (reference number 029/08).

### 3.1. Primary PDL Cell Isolation and Culture

PDL tissues were obtained from the middle third of the root surface of caries-free teeth, extracted during routine orthodontic procedures from periodontally healthy donors. The tissues were cultured in T75 cell culture flasks (CELLSTAR^®^ Greiner BioOne, Kremsmünster, Austria) using N2B27-PDLsf medium [23] at 37 °C in a humidified atmosphere with 5% CO_2_. Cells were passaged upon reaching confluence, with the medium supplemented with 1% Penicillin/Streptomycin (Gibco, Carlsbad, CA, USA) and 1% Plasmocin prophylactic (Invivogen, Toulouse, France) until passage 2. From passage 3 onward, the media were used without Penicillin/Streptomycin and Plasmocin prophylactic.

Cells expanded to passages 3 and 4 were employed for subsequent analyses. Passaging was performed using StemPro Accutase (Gibco) for 5–10 min at 37 °C, with enzyme activity neutralized by diluting with culture medium.

### 3.2. Mechanical Loading of PDL Cells

Static tensile strain was applied to PDL cells seeded at a density of 20,000 cells per well in 2 mL of culture medium. The cells were grown to 80% confluence on Bioflex^®^ culture plates, which were coated with type I collagen and featured flexible silicone membrane-bottom wells designed to facilitate mechanical stimulation (BF-3001C; Flexcell International, Hillsborough, NC, USA). The plates were placed into a strain device (FX-6000T™ Tension System, Flexcell International), which includes a BioFlex baseplate with a cylindrical post as the loading platform, matching the dimensions of the flexible-bottom wells. This system employs a computer-regulated bioreactor that utilizes vacuum and positive air pressure to deliver precisely controlled, static deformation to cells growing in a monolayer. Following cell seeding and a 24 h growth period, continuous stretching of the cells was performed at 2.5% (1.3 cN/mm^2^), 5% (2.6 cN/mm^2^), 10% (5.2 cN/mm^2^), and 20% strain (10.4 cN/mm^2^) [24,25,26]. The BioFlex baseplate, along with the loading stations and posts, was then placed in an incubator to maintain a humidified atmosphere with 5% CO_2_ at 37 °C. Cells were subjected to mechanical loading for 24 h. After this period, the plates were removed, and the flexible membranes with the stretched cells were prepared for further experimentation. To elucidate the mechanisms triggered by tension-induced mechanical loading, unstretched cells were used as controls in each experiment.

### 3.3. RNA Extraction, Quality Control and cDNA Synthesis

Total messenger ribonucleic acid (mRNA) was isolated and purified from cell lysates using the RNeasy Mini Kit (Qiagen, Hilden, Germany) in accordance with the manufacturer’s instructions. The concentration of the isolated mRNA was determined spectrophotometrically using a Nanodrop (Thermo-Fisher Scientific, Waltham, MA, USA), and its purity was assessed by the 260/280 absorbance ratio. Subsequently, mRNA was reverse-transcribed into complementary deoxyribonucleic acid (cDNA) using the iScript Select cDNA Synthesis Kit (Bio-Rad Laboratories, Hercules, CA, USA). The 20 µL cDNA synthesis reaction was prepared with oligo(dT) primer mix following the manufacturer’s protocol. Each reaction utilized the maximum available amount of RNA from each sample, with a maximum of 1 µg total RNA per reaction. The synthesis process involved an initial cDNA synthesis step at 42 °C for 90 min, followed by reverse transcriptase inactivation at 85 °C for 5 min, both conducted using an iCycler (Bio-Rad Laboratories).

### 3.4. RT-qPCR to Detect Transcriptional Changes in PDL Cells Exposed to Strain

qPCR was performed using Faststart universal SYBR Green (Sigma-Aldrich, St. Louis, MO, USA) and a StepOnePlus Real-Time PCR System (Thermo-Fisher Scientific). mRNA values were normalized to the GAPDH housekeeping gene, which is constitutively expressed at high levels, to account for general variability in mRNA expression between samples. Genes that were within 12 cycles of the cycle at which GAPDH reached the threshold for expression were considered expressed in the PDL cells. Primer sequences are presented in Table 2 including catalogue numbers from Origene.

### 3.5. In Vivo Preparation of Histological Specimens of Teeth With and Without OTM Exposure

For in vivo analyses, teeth with adjacent PDL were obtained from adolescent patients initiating orthodontic treatment with fixed multibracket appliances and who required symmetric premolar extractions in one jaw. The selection criteria for participants included overall good health, absence of medications influencing bone or soft tissue metabolism, no prosthetic restorations on the teeth to be moved, absence of premature occlusal contacts, no radiographic evidence of horizontal bone loss or vertical bony defects, and no signs of root resorption. Due to the inherent tendency of brackets to act as reservoirs for plaque accumulation, it was essential that all participants strictly adhere to comprehensive oral hygiene protocols. These included brushing after every meal, using interdental brushes, and following detailed instructions on the proper technique for brushing bracketed teeth, which were provided prior to bracket placement. Furthermore, adherence to proper oral hygiene was regularly monitored and reinforced at each follow-up appointment to ensure consistent and effective oral care throughout the duration of the treatment. This precaution was necessary to prevent plaque-induced bacterial inflammation and ensure the reliability of the study results. In the context of the multibracket appliance insertion, one of the two premolars designated for extraction was fitted with a bracket for biomechanical purposes, thereby facilitating optimal movement of the adjacent teeth, and conversely, the contralateral premolar was left unbracketed. Consequently, the bracketed premolar experienced orthodontic loading during the leveling phase, while the unbracketed premolar served as a reference for physiological loading without OTM. Following a period of 3–8 weeks, determined by the individual therapeutic needs, both premolars were extracted to create space for therapeutic purposes and were immediately processed for subsequent analyses.

Post-extraction, the teeth were preserved by immersion in 4% buffered formaldehyde (Sörensen buffer) at room temperature for a minimum of 24 h, followed by decalcification in 4.1% disodium ethylenediaminetetraacetic acid (EDTA) solution for at least one month, with the solution being refreshed every 24 h. After hydration, the specimens were dehydrated through an ascending series of ethanol concentrations, embedded in paraffin, and serial sagittal sections of 2–3 µm were prepared for further examination.

### 3.6. Immunostaining

To visualize the presence of primary cilia, primary human PDL cells were seeded at a density of 10,000 cells per coverslip in 1 mL of culture medium and maintained on glass-bottom dishes (Marienfeld Laboratory Glassware, Lauda-Königshofen, Germany) for a period of five consecutive days. On each day, cells were fixed with paraformaldehyde (PFA, Sigma-Aldrich, St. Louis, MO, USA) prior to incubation with the primary antibody against acetylated α-tubulin at 4 °C overnight. Bound antibodies were detected using fluorescent secondary antibody Alexa Fluor 568, applied at room temperature for 1 h. Nuclei were counterstained with DAPI for 10 min.

Images were acquired using a fluorescent microscope (Keyence BZ-X710, Keyence, Ōsaka, Japan). Laser lines at 405 nm and 561 nm were employed for sample excitation, with settings held constant across all analyzed images. To examine primary cilia and BMP signaling with tooth movement in vivo, histology sections of human teeth were deparaffinized, rehydrated, and rinsed for 10 min in tris-buffered saline (TBS). Following this, the sections were permeabilized with 0.1% Tween 20 (Sigma, Aldrich) for 5 min at room temperature, blocked with 10% goat serum (Sigma Aldrich) for 1 h at room temperature, and incubated in primary antibodies at 4 °C overnight. Antibodies against pSMAD1/5/8 (rabbit polyclonal, 1:250, Fisher Scientific, NH, USA) and acetylated α-tubulin (mouse monoclonal, 1:10, Sigma-Aldrich) were used to visualize BMP signaling and primary cilia, respectively. Sections were then incubated in the secondary antibodies anti-mouse Alexa Fluor 488 (1:500, Life Technologies, Carlsbad, CA, USA) and anti-rabbit Alexa Fluor 568 (1:500, Life Technologies) at room temperature for 1 h. Stained slides were then mounted with media containing the nuclear stain DAPI and sealed with nail polish (Electron Microscopy Sciences, Hatfield, PA, USA). Images were captured using a 316 Discover ECHO Confocal Microscope (ECHO, San Diego, CA, USA). The validity of the assays was ensured by routinely performing negative controls to exclude any artifacts, obtained by replacing the primary antibody with TBS/BSA. All antibodies were utilized at previously optimized concentrations. The quality of the original microphotographs of the stainings was routinely verified for high resolution and clarity, with particular attention to the coloring, to ensure no reduction in image quality.

### 3.7. Statistical Analysis

Differences between control and experimental groups were determined using a two-tailed Student’s t-test assuming equal variance. Values are reported as mean ± SEM, with *p* < 0.05 considered statistically significant. The sample size was selected to achieve a power of at least 80%. Experimental groups in transcriptional analyses were expressed as a fold change in relation to static controls normalized to a value of “1”. Statistical analysis was conducted using GraphPad Prism (San Diego, CA, USA).

## 4. Discussion

This study is the first to investigate the role of primary cilia in human periodontal PDL cells under orthodontic force, specifically exploring their involvement in mechanotransduction and the modulation of BMP signaling, a pivotal pathway in craniofacial tissue development and maintenance. While primary cilia are well documented for their involvement in mechanotransduction and cellular homeostasis across various tissues, their role in PDL cells during OTM remained largely unexplored so far. The research presented here fills this knowledge gap by examining the presence, structure, and function of primary cilia in PDL cells and their potential interaction with BMP signaling pathways.

The findings of this investigation confirmed the presence of primary cilia in human PDL cells both in vitro and in vivo and revealed that their structural characteristics such as length and incidence are dynamically regulated in response to mechanical strain and culture conditions such as cellular density. Previous studies have demonstrated that the length of primary cilia is a critical determinant of their ability to detect mechanical stimuli, with elongation enhancing their sensitivity to such forces [27]. It is well established that inflammatory cytokines and other signaling molecules influence cilia formation and elongation, which is particularly relevant in the context of orthodontic treatment, where mechanical forces induce sterile inflammation within periodontal tissues, potentially heightening the mechanosensitivity of PDL cells [10].

Transcriptional analyses of PDL cells exposed to orthodontic forces revealed the expression of key components of the BMP signaling pathway, with several candidates emerging as potential facilitators of mechanosensation. Notably, the in vivo data provide compelling evidence for the involvement of primary cilia in mediating mechanotransduction during OTM. These results suggest that primary cilia may enhance BMP signaling, which is consistent with similar mechanisms observed in other cell types under mechanical stimulation, though this represents a novel discovery within the PDL [20,22]. Activation of BMP signaling in response to OTM coincided with a significant increase in primary cilium incidence and elongation in this study, particularly in regions where BMP signaling was activated. This supports the hypothesis that primary cilia may serve as critical mediators in the transduction of mechanical signals through the BMP pathway.

While primary cilia were detected in the PDL of both moved and static teeth, not all PDL cells exhibited a primary cilium. This finding is consistent with the dynamic nature of primary cilia, which can shorten or undergo disassembly in the absence of external stimuli [15]. This could explain the lower incidence of primary cilia in the PDL of static teeth, where mechanical forces are not applied. Additionally, the random orientation of primary cilia in the PDL may have contributed to some projections not being captured in the tissue sections. In contrast, increased primary cilium incidence and elongation were observed in the PDL of moved teeth, particularly in regions where BMP signaling was activated.

Further analysis of Ift88, a key ciliary marker, revealed that expression levels varied with mechanical strain. Specifically, Ift88 expression was elevated at low strain levels (2.5% and 5%), suggesting a threshold for axoneme elongation and cilium adaptation. These results align with previous research indicating that primary cilia are more prevalent on cells subjected to low mechanical forces and possess heightened sensitivity to these subtle strains [20,22]. Conversely, exposure to higher mechanical strains has been shown to disrupt ciliary architecture, with many cells subjected to these forces lacking primary cilia altogether [28,29].

The transcriptional analysis of PDL cells subjected to tensile strain further elucidated the role of BMP signaling in the mechanotransduction process. Previous studies have demonstrated that endothelial cell primary cilia enhance BMP9-Smad1/5/8 signaling exclusively under low strain intensities [22]. The BMP–Alk–Smad signaling axis is a conserved mechanism for mechanosensing, with its activity strongly dependent on the magnitude of applied force [20]. In our study, we observed a variable transcriptional response to BMP signaling components across different strain conditions, with a trend of increased BMP9 expression under higher strain levels. Analysis of key BMP signaling molecules, including Smad1, Smad5, Smad8, and Smad9, revealed a heterogeneous response to mechanical strain, characterized by both upregulation and downregulation. Notably, Smad1 showed a pronounced downregulation in response to higher strain intensities, suggesting a potential inhibitory or compensatory role aimed at preventing excessive inflammatory responses or immune reactions.

Type I BMP receptors exhibited consistent upregulation across all strain conditions, though inter-donor variability suggests a complex and cell-specific regulatory response. Type II BMP receptors were upregulated exclusively under the highest strain condition (20%), indicating a strain-specific regulatory pattern. These observations emphasize the need to consider the cellular context when interpreting BMP signaling responses to mechanical stimuli. The sustained upregulation of Bmpr2, Bmp7, and Alk2 across all strain conditions suggests that these components may play a crucial role in the PDL cell response to mechanical loading, possibly contributing to the adaptive cellular mechanisms that preserve homeostasis and modulate host responses to prevent excessive immune reactions.

Despite these novel insights, several limitations of the study design must be acknowledged. First, while this investigation identified key interactions between primary cilia and BMP signaling in mechanotransduction, further research is needed to determine whether primary cilia directly interact with BMP signaling components in PDL cells. Second, the observed transcriptional patterns suggest that BMP signaling and ciliary regulation are highly context-dependent, complicating the ability to draw generalized conclusions across different strain conditions. Although a 24 h strain protocol was used to simulate OTM, it is possible that shorter durations of mechanical loading could yield more precise insights into cilia dynamics and BMP signaling. For example, other cell types have shown that primary cilia undergo negative feedback mechanisms within hours of stimulation, and BMP signaling activation often plateaus after initial induction.

Additionally, while the in vitro model focuses on acute responses to mechanical strain, it does not address the long-term adaptations of PDL cells to sustained orthodontic forces, which are crucial for understanding chronic immune responses and potential pathological conditions during orthodontic treatment. These limitations highlight the need for future research to explore the temporal dynamics of primary cilia and BMP signaling and to investigate the long-term mechanotransductive responses of PDL cells to orthodontic forces.

In conclusion, this study underscores the complex interplay between mechanical forces, cellular signaling, and structural adaptations in PDL cells. The differential gene expression patterns observed in response to varying strain intensities emphasize the intricate nature of mechanotransduction in the PDL, with primary cilia likely serving as central mediators of this process. Future research should focus on elucidating the temporal dynamics of primary cilia and BMP signaling activation, as well as exploring their interactions with other mechanosensitive pathways, such as Wnt/β-catenin signaling. The clinical implications of this study are particularly relevant to the prevention of ERR during OTM as a common complication associated with prolonged mechanical loading. ERR can occur as a result of the inflammatory processes triggered by orthodontic forces, and it is often linked to excessive or improper loading that disrupts the normal remodeling of the PDL. Given that primary cilia are implicated in mechanotransduction and cellular signaling, particularly in response to mechanical strain and BMP signaling pathways, understanding their role in PDL cells provides a potential avenue for mitigating this complication. Given the dynamic regulation of primary cilia length and incidence in response to mechanical strain, as observed in this study, therapeutic strategies aimed at optimizing ciliary function could potentially prevent excessive inflammatory responses that contribute to root resorption. For instance, modulating the activity of BMP receptors or promoting primary cilium elongation under low strain conditions could enhance the mechanosensitive capacity of PDL cells, thus promoting a more controlled and beneficial remodeling process. Additionally, targeting the pathways that influence ciliary formation, such as inflammatory cytokine signaling, might provide a means of reducing the risk of unwanted resorptive activity on the root surface. Incorporating cilia-based therapies or interventions into orthodontic treatment could therefore provide clinicians with a more refined approach to managing mechanical forces. By ensuring that PDL cells respond appropriately to strain via enhanced mechanotransduction and BMP signaling, it may be possible to prevent or limit the extent of ERR, ultimately improving patient outcomes and the long-term success of orthodontic treatment. Future studies should focus on validating these potential therapeutic strategies, exploring the effects of modulating primary cilia in clinical settings, and determining how these interventions can be applied to prevent ERR during orthodontic treatment.

## Figures and Tables

**Table 1:** Trends in mRNA expression of primary human PDL cells under static conditions and with exposure to tensile strain.

Trends in Gene Expression: Summary of Cells Isolated from 4 Patients					
Gene	Expression	2.5% Strain	5% Strain	10% Strain	20% Strain
*Cox2*	Yes			Increase	Increase
*Ift88*	Yes	Increase	Increase		
*Id1*	Yes				
*Id3*	Yes				
*Smad1*	Yes			Decrease	Decrease
*Smad5*	Yes				Increase
*Smad8/9*	Yes	Increase	Increase	Decrease	Increase
*Bmp2*	Yes	Increase			
*Bmp4*	Yes				Increase
*Bmp7*	Yes	Increase	Increase	Increase	Increase
*Bmp9*	Yes			Increase	Increase
*Alk1*	Yes			Increase	Increase
*Alk2*	Yes	Increase	Increase	Increase	Increase
*Alk3*	Yes				
*Alk4*	No				
*Alk5*	Yes	Increase	Increase		
*Alk6*	Yes		Increase	Increase	
*Alk7*	Yes				Increase
*Bmpr2*	Yes	Increase	Increase	Increase	Increase
*Acvr2a*	Yes				Increase
*Acvr2b*	Yes				Increase
*Tgfbr2*	Yes				Increase

**Table 2:** Human primer sequences for RTqPCR.

Gene	Forward Primer	Reverse Primer	Origene Catalog #
ACVR2A	GCCAGCATCCATCTCTTGAAGAC	GATAACCTGGCTTCTGCGTCGT	HP205440
ACVR2B	CGCTTTGGCTGTGTCTGGAAGG	CAGGTTCTCGTGCTTCATGCCA	HP204836
ALK1	GCGACTTCAAGAGCCGCAATGT	TAATCGCTGCCCTGTGAGTGCA	HP200007
ALK2	GACGTGGAGTATGGCACTATCG	CACTCCAACAGTGTAATCTGGCG	HP230471
ALK3	CTTTACCACTGAAGAAGCCAGCT	AGAGCTGAGTCCAGGAACCTGT	HP207659
ALK4	TCAGAAGCTGCGTCCCAACATC	CGTTGGCATACCAACACTCTCG	HP207638
ALK5	GACAACGTCAGGTTCTGGCTCA	CCGCCACTTTCCTCTCCAAACT	HP207900
ALK6	CTGTGGTCACTTCTGGTTGCCT	TCAATGGAGGCAGTGTAGGGTG	HP205133
ALK7	TGCTAGTGGTCTGGCACACCTT	CTTCACAGCCAACCCTAAGTCC	HP233859
BMP2	TGTATCGCAGGCACTCAGGTCA	CCACTCGTTTCTGGTAGTTCTTC	HP205130
BMP4	CTGGTCTTGAGTATCCTGAGCG	TCACCTCGTTCTCAGGGATGCT	HP205132
BMP7	GAGTGTGCCTTCCCTCTGAACT	AGGACGGAGATGGCATTGAGCT	HP205524
BMP9	CATTGTGCGGAGCTTCAGCATG	CTGGTGATCTGCTCATGCCTAG	HP212042
BMPR2	AGAGACCCAAGTTCCCAGAAGC	CCTTTCCTCAGCACACTGTGCA	HP205134
COX2	CGGTGAAACTCTGGCTAGACAG	GCAAACCGTAGATGCTCAGGGA	HP200900
GAPDH	GTCTCCTCTGACTTCAACAGCG	ACCACCCTGTTGCTGTAGCCAA	HP205798
ID1	GTTGGAGCTGAACTCGGAATCC	ACACAAGATGCGATCGTCCGCA	HP205903
ID3	CAGCTTAGCCAGGTGGAAATCC	GTCGTTGGAGATGACAAGTTCCG	HP205905
IFT88	TCGGCTAGATGAGGCTTTGGAC	CACTGACCACCTGCATTAGCCA	HP227511
OPN	CGAGGTGATAGTGTGGTTTATGG	GCACCATTCAACTCCTCGCTTTC	HP200549
SERPINE1	CTCATCAGCCACTGGAAAGGCA	GACTCGTGAAGTCAGCCTGAAAC	HP200569
SMAD1	TTGGCACAGTCTGTGAACCATGG	GTAACATCCTGGCGGTGGTATTC	HP228495
SMAD2	GGGTTTTGAAGCCGTCTATCAGC	CCAACCACTGTAGAGGTCCATTC	HP231074
SMAD3	TGAGGCTGTCTACCAGTTGACC	GTGAGGACCTTGTCAAGCCACT	HP208949
SMAD5	CAGGAGTTTGCTCAGCTTCTGG	GGTGCTGGTTACATCCTGCCG	HP226654
SMAD8/9	GTGCTGTGAGTTCCCATTTGGC	TTCACTGTGTCTTGGCACGAGC	HP208951
TGFB1	TACCTGAACCCGTGTTGCTCTC	GTTGCTGAGGTATCGCCAGGAA	HP200609
TGFBR2	GTCTGTGGATGACCTGGCTAAC	GACATCGGTCTGCTTGAAGGAC	HP233478

## Data Availability

The raw data supporting the conclusions of this article will be made available by the authors on request.
